# Augmentation of Dermal Wound Healing by Adipose Tissue-Derived Stromal Cells (ASC)

**DOI:** 10.3390/bioengineering5040091

**Published:** 2018-10-26

**Authors:** Joris A. van Dongen, Martin C. Harmsen, Berend van der Lei, Hieronymus P. Stevens

**Affiliations:** 1 Department of Pathology & Medical Biology, University of Groningen and University Medical Centre of Groningen, 9713 GZ Groningen, The Netherlands; jorisavandongen@gmail.com (J.A.v.D.); m.c.harmsen@umcg.nl (M.C.H.); 2 Department of Plastic Surgery, University of Groningen and University Medical Centre of Groningen, 9713 GZ Groningen, The Netherlands; info@berendvanderlei.nl; 3 Department of Plastic Surgery, Bergman Clinics, Den Haag & Rijswijk, Binckhorstlaan 149, 2516 BA Den Haag, The Netherlands

**Keywords:** adipose derived stromal cells, lipografting, wound healing, stem cells, stromal vascular fraction, skin

## Abstract

The skin is the largest organ of the human body and is the first line of defense against physical and biological damage. Thus, the skin is equipped to self-repair and regenerates after trauma. Skin regeneration after damage comprises a tightly spatial-temporally regulated process of wound healing that involves virtually all cell types in the skin. Wound healing features five partially overlapping stages: homeostasis, inflammation, proliferation, re-epithelization, and finally resolution or fibrosis. Dysreguled wound healing may resolve in dermal scarring. Adipose tissue is long known for its suppressive influence on dermal scarring. Cultured adipose tissue-derived stromal cells (ASCs) secrete a plethora of regenerative growth factors and immune mediators that influence processes during wound healing e.g., angiogenesis, modulation of inflammation and extracellular matrix remodeling. In clinical practice, ASCs are usually administered as part of fractionated adipose tissue i.e., as part of enzymatically isolated SVF (cellular SVF), mechanically isolated SVF (tissue SVF), or as lipograft. Enzymatic isolation of SVF obtained adipose tissue results in suspension of adipocyte-free cells (cSVF) that lack intact intercellular adhesions or connections to extracellular matrix (ECM). Mechanical isolation of SVF from adipose tissue destructs the parenchyma (adipocytes), which results in a tissue SVF (tSVF) with intact connections between cells, as well as matrix. To date, due to a lack of well-designed prospective randomized clinical trials, neither cSVF, tSVF, whole adipose tissue, or cultured ASCs can be indicated as the preferred preparation procedure prior to therapeutic administration. In this review, we present and discuss current literature regarding the different administration options to apply ASCs (i.e., cultured ASCs, cSVF, tSVF, and lipografting) to augment dermal wound healing, as well as the available indications for clinical efficacy.

## 1. Introduction

The skin is a physical barrier and the largest self-repairing organ of the human body and serves multiple physiological functions, such as protection against dehydration and thermal, chemical, or physical stress [1,2]. Repair of physical skin damage requires effective wound healing, which is a dynamic process that involves five overlapping stages: homeostasis, inflammation, proliferation, re-epithelization, and finally fibrosis [3]. These stages of wound healing are regulated by platelets and immune cells, such as monocytes and neutrophils, which secrete chemokines and cytokines that attract and instruct other locally present cell types in the skin [4,5,6,7]. The skin consists of multiple layers starting from the outside with the epidermis (consist of stratum corneum, -lucidum (only present in hairless skin), -granulosum, -spinosum, and -basale), dermis, and a subcutaneous layer (a layer of adipose tissue containing adipocytes embedded in the stromal vascular fraction (SVF)). The epidermis is organized into hair follicles containing the interfollicular epidermis as well as sebaceous glands. The interfollicular epidermis is maintained by hair follicle stem cells and associated progenitor cells, which are also responsible for hair regrowth [8,9]. After physical damage of the epidermis, hair follicle stem cells migrate to the wound area to regenerate this damaged tissue [9]. Besides hair follicle stem cells and associated progenitor cells, the epidermis consists mainly of keratinocytes in various stages of differentiation, starting at the stratum basale as basal keratinocytes [3]. During life, basal keratinocytes continuously migrate upwards to the stratum corneum. At the base of the stratum corneum, keratinocytes undergo apoptosis, which adds to layers of the stratum corneum and functions to establish and maintain the physical barrier of the skin compromising environmental factors [3]. The dermis comprises vasculature and nerves, as well as specialized extracellular matrix. In this, elastin and collagen are pivotal, which provide, respectively, elasticity and tear-resistance to the dermis [10]. Finally, the subcutaneous layer comprises adipocytes and SVF. The SVF consist of all non-parenchymal (adipocyte) cell types i.e., fibroblasts, immune cells, endothelial cells, pericytes, and adipose tissue-derived stromal cells (ASCs) [11,12].

Cultured ASCs secrete a plethora of angiogenic, anti-fibrotic, and anti-apoptotic growth factors [13,14,15]. The isolation and characterization of ASCs from lipoaspirates was first described by Zuk et al. in 2001 [13]. The possibility to isolate ASCs resulted in an increased rate of publications that described the behavior and regenerative effects of ASCs with regard to important processes related to dermal wound healing, such as angiogenesis and fibroblast migration [16,17]. Thus far, however, the use of ASCs as a cellular therapy for dermal wound healing has only obtained the status of a clinical experimental treatment modality. Although there are several reports and studies indicating a regenerative effect of the use of ASCs, sound scientific evidence is lacking: there are no prospective randomized clinical trials that support the postulated influence of ASCs. Moreover, the harvesting and processing procedures of ASCs are highly variable while randomized clinical trials warrant uniform procedures. Firstly, a single-cell suspension of ASCs can only be efficiently isolated enzymatically. The use of enzymes, such as collagenase, to disassemble intact adipose tissue or lipoaspirates to prepare a cell suspension-based SVF (cSVF) is legally forbidden in many countries. Secondly, intraoperative enzymatic isolation procedures are time-consuming and expensive [12]. In clinical perspective, the most relevant hurdle of enzymatic methods is the poor retention of cells after administration of single-cell suspensions of ASCs. This is relevant in skin applications, in which the ubiquitous lymphatics may rapidly drainage via lymphatics that are abundantly present in skin, in particular, in wound beds [18]. Nowadays, it is proven that also ASCs can be found concentrated in SVF that is mechanically isolated from adipose tissue while these procedures are significantly simpler and faster than enzymatic procedures [12].

In this overview, we present current literature regarding the different options to administrate ASCs (i.e., cultured ASCs, SVF, and lipografting) to augment dermal wound healing, as well as the thus far available indications for clinical efficacy.

## 2. Adipose Derived Stromal Cells as Cellular Therapy for Dermal Wound Healing

In dermal wounds, the parenchymal tissue of the skin is damaged and needs to be remodeled in order to regenerate tissue. Regeneration of damaged parenchymal tissue is postulated to be under the control of autologous stromal cells i.e., ASCs. ASCs are mesenchymal stromal cells that are present in SVF of adipose tissue, attached around vessels as pre-cursor cells (i.e., pericytes and periadventitial cells) [19,20]. In vitro, ASCs have the ability to differentiate into multiple cell lineages, like ectodermal, endodermal, as well as mesenchymal lineages [13,21,22]. Despite their lack of self-renewal, their differentiation capacity persuaded investigators to name ASCs adipose derived stem cells [23,24,25,26]. In vitro, ASCs have a limited lifespan, with a limited proliferation potential, and undergo senescence at higher passages. Nevertheless, this is probably irrelevant for therapeutic use of ASCs for wound healing because irrespective of proliferation, ASCs are able to differentiate (be constructive), secrete growth factors and cytokines (be instructive), and can remodel the extracellular matrix (be reconstructive) [27].

To date, two clinical trials directed at safety of revascularization after critical limb ischemia, have investigated the use of cultured ASCs for critical limb ischemia (Table 1) [28,29]. In both studies, cultured ASCs were injected intramuscularly to treat patients with non-healing ischemic ulcers. In twelve patients the response rate of ulcer healing was 66.7% of the patients after six months [29]. Moreover, an overall decrease in pain and improved walking distance in claudication as compared to the baseline was noted after six months. The long-term outcome is unknown unfortunately. In the other trial, only seven patients were treated, of which four patients underwent amputation within five months after injection of ASCs [28]. Three non-amputated patients reported a decrease of pain six months postoperative. Besides the amputations, no serious complications were reported in both studies (Table 1) [28,29]. The improved ulcer healing, at least partly, appears to relate to the augmented angiogenesis by the administered ASCs. Although the results from these non-controlled, small-scaled studies are promising for part of the treated patients, the therapeutic effect needs to be corroborated in randomized, placebo-controlled large trials. This is essential, if alone to distinguish responders from non-responders and to determine optimal dosing, time-to-treat, frequency of dosing, as well as to identify parameters that dictate efficient and effective wound healing in adipose tissue or its constituents.

Large proportion of patients did not respond after administration of cultured ASCs. This lack of effect of ASCs might relate to disturbed migration and/or disposal of ASCs via the circulation and lymph system within the first 24 h after injection [18]. Other factors that influence the therapeutic impact of ASCs are donor characteristics (e.g., age or co-morbidity, such as diabetes mellitus), as well as the induction of senescence of ASCs after enzymatic isolation and culture [30,31,32]. The two studies on the therapeutic benefit of ASCs on critical limb ischemia, however, showed that age or co-morbidity did not influence the therapeutic impact of ASCs [28,29]. Senescence occurred when cells are cultured more than ten passages [32]. Senescent cells do not proliferate or differentiate while these are pro-inflammatory and exhibit increased production of reactive oxygen species (ROS) [33,34]. This senescent status is referred to as the senescence-associated secretory phenotype (SASP), which impacts surrounding tissue cells. ROS accumulation inhibits proliferation and proangiogenic capacities of ASCs which impairs their wound healing support [35]. Hyperglycemia that is associated with diabetes may cause ROS accumulation in ASCs. Although we have shown that ASCs are refractory to chronic hyperglycemia, their sensitivity to acute hyperglycemia is high [36,37]. Lee et al. used ASCs in passage 3, while Bura et al. did not mention the used passage number [28,29]. Furthermore, ASCs undergo phenotypic changes, other than senescence, upon several passages of culture. During culture the phenotype and function of ASCs emerges, e.g., secretion of a plethora of cytokines and growth factors, matrix metalloproteinases and extracellular matrix [19,38,39]. In vitro, ASCs are a typical culture artefact that differs from their in vivo peers, which is an ill-conceived fact. Therefore, the direct translation of in vitro characteristics of ASCs to their physiological function in adipose tissue or SVF falls short of adequate scientific evidence. Therefore, the use of ASCs in their natural habitation with their original function i.e., SVF or lipografting might result in a different clinical outcome in comparison to cultured ASCs. This, however, does not preclude the study of the therapeutic characteristics and benefits of cultured ASC.

## 3. Cellular or Tissue Stromal Vascular Fraction as Treatment for Dermal Wound Healing

The legal ban on enzyme use to isolate SVF or ASCs is based on hypothetical increased risk of animal-derived products, while these multistep procedures are considered undesirable manipulations. Therefore, new intraoperative isolation procedures without the use of enzymes and animal derived products have been developed, the so-called mechanical isolation procedures [12]. Mechanical isolation procedures yield essentially intact tissue that is devoid of adipocytes, hence our proposed term: tissue stromal vascular fraction (tSVF). In tSVF, the intact extracellular matrix with all of its bound regenerative trophic factors, maintains the integrity of the stromal cells too [12,40]. In contrast, enzymatic isolation procedures yield a SVF that comprises a suspension of cells (cSVF) that obviously lack intercellular connections and extracellular matrix (ECM). The ECM is an important reservoir of regenerative growth factors while ECM molecules and their degradation products also contribute to the regenerative power of the ECM [12,41,42]. The predicted therapeutic capacity of cSVF, therefore, is lower than of tSVF, which remains to be assessed in side by side comparative studies. However, future therapies might comprise hybrids in which ECM is used to deliver and retain ASCs in the wounds after injection. In this way, the ECM instructs ASCs to differentiate and secrete their trophic factors to induce angiogenesis, remodel the native extracellular matrix, and reduce inflammation for a longer time as compared to a single cell injection of ASCs [15,43]. Finally, ECM components regulate proliferation and migration of cells as well as angiogenesis. An adequate juxtracrine communication between different cell types e.g., pericytes and endothelial cells augments angiogenesis [39]. Angiogenesis might even be further stimulated by the vasculature in tSVF, which would result in reduced ischemia and thus reduced apoptosis [44]. This improves graft take and survival of the transplanted tSVF as well as augmented dermal wound healing.

To date, several clinical studies report the use of cSVF to increase dermal wound healing (Table 2) [45,46,47,48,49]. As of yet, none of these studies used tSVF as control or as experimental treatment. In total, seventy-three patients with ulcers were treated with cSVF, of which sixty-three showed complete healing. One study mixed cSVF with fibrinogen and thrombin and compared this with only fibrinogen and thrombin after applying to diabetic ulcers [47]. 62% of the patients showed complete healing after treatment with fibrinogen and thrombin. 100% of the patients showed complete healing of the diabetic ulcers after treatment with cSVF in combination with fibrinogen and thrombin [47]. All studies, except the aforementioned study, also showed a decrease in wound-associated pain after injection of cSVF. In one prospectively controlled study, ten patients with peripheral arterial disease and chronic non-healing ulcers were treated with cSVF [45]. As a control, ten patients received no treatment. This study showed that six out of ten patients presented complete ulcer healing while four patients did not respond to the treatment [45]. No complications were reported in any study (Table 2). These studies show that cSVF seems to be partly effective in increasing wound healing rates after injection. Complete ulcer healing in all patients only occurred when fibrinogen, an extracellular matrix protein, and thrombin was added to cSVF, suggesting that the use of tSVF might be more effective in closing dermal wounds as compared to cSVF. After activation with thrombin, fibrinogen forms a dense fibrin network that entraps the cells in cSFV, which mimics a native stromal tissue structure.

## 4. Lipografting as a Treatment for Dermal Wound Healing

Lipografting, the transplantation of lipoaspirates i.e., fragmented adipose tissue, is widely investigated in several clinical studies as a treatment to promote dermal wound healing [50,51,52,53,54,55,56,57]. Three prospective studies used lipografting in combination with platelet rich plasma (PRP) or cSVF to treat lower extremity ulcers in ninety patients [53,54,55]. Cervelli et al., used PRP as additive to improve cellular growth and differentiation of cells present in the lipograft as well as recipient cells [55]. As a control, collagen and hyaluronic acid was used to treat lower extremity ulcers and vascular disease. After 9.7 weeks on average, the ulcers of sixteen out of twenty patients (80%) had re-epithelialized in the experimental group, while ulcers in five out of ten patients (50%) had re-epithelialized in the control group after 8.4 weeks on average [55]. Although these differences suggest a therapeutic impact of lipografts, the numbers were too low to allow for statistical significance. In a later study, these authors treated patients with ulcers (n = 30) with PRP-enriched lipografts and showed complete ulcer healing in 57% of the patients after three months [53]. In another follow-up, the same authors compared cSVF enriched lipografting with hyaluronic acid as well as PRP-enriched lipografting with bare PRP to treat posttraumatic lower extremity ulcers in forty patients [54]. This study showed similar re-epithelialization rates for the cSVF enriched lipografting and PRP enriched lipografting groups, respectively 97.9% ± 1.5% and 97.8% ± 1.5% after 9.7 weeks. The control groups showed similar re-epithelialization rates as well with 87.8% ± 4.4% for the hyaluronic acid and 89.1% ± 3.8% for the PRP group, which were significantly lower than for the cSVF and PRP enriched lipografting groups (*p* < 0.05) [54]. These studies by the Cervelli group indicate that lipografting enriched with cSVF or PRP promotes dermal wound healing. However, the comparison of cSVF or PRP enriched lipografting to only lipografting, is required to assess the influence and necessity of the addition of cSVF or PRP to lipografts in ulcer treatments. 

Four other studies described the use of non-supplemented lipografts to treat non-healing ulcers (n = 36). Two of these studies were case reports while none of the four studies had a control group (Table 3). Van Abeelen et al. treated one patient with recurrent leaks from stoma and skin excoriations with lipografting and prevented the stoma from leaking [50]. Another case report by Caviggioli et al. used lipografting as a treatment for a posttraumatic leg ulcer in one patient. After one month, the posttraumatic ulcer was closed [52]. One study was a prospective study by Stasch et al. and described that lipografting augmented the complete healing of twenty-two out of twenty-five non-healing diabetic ulcers, while one patient needed an additional lipografting session to finally close the diabetic ulcer as well [57]. Klinger et al. retrospectively analyzed eight patients with posttraumatic scars in combination with chronic ulcers and showed complete re-epithelialization after lipografting [56]. Yet, without the comparison of lipografting with a placebo group the effect of lipografting on dermal wound healing remains unclear. In all seven studies, a few minor complications occurred, and, therefore, lipografting to promote dermal wound healing appears a relatively safe procedure (Table 3).

The clinical efficacy of lipografting is usually ascribed to ASCs in the stromal vascular fraction, more than to the adipocytes that comprise most of the lipograft’s volume. However, the ischemia that occurs in lipoaspirates upregulates regenerative factors, such as fibroblast growth factors and vascular endothelial growth factor, in particular in adipocytes. These growth factors promote angiogenesis and proliferation of skin cells while these suppress apoptosis, thus could contribute to the wound repair effect of lipografts [14]. Alternatively, one could envisage lipografting as a method to deliver ASCs to the damaged skin to kick-start regeneration. This does not preclude that other regenerative cells in adipose tissue such as the precursors of ASCs contribute to the therapeutic benefit of lipografts. Moreover, the administration of ASCs as present in cSVF or tSVF, might be more beneficial in the early phase of healing of chronic wounds because only a small volume is required. In this phase, cells with regenerative capacity rather than large volume cells i.e., adipocytes, are warranted. In this way, (small) chronic wounds can be treated already in the early phase which also restricts the risk of infection.

## 5. Conclusions

The administration of ASCs is promising as new therapy for the treatment of non-healing dermal wounds. There is a variety of formulations of ASCs that can be injected i.e., as cultured cells, cSVF, tSVF, or lipografts to augment dermal wound healing. However, due to the lack of well-designed randomized placebo-controlled clinical trials, none of these formulations can be designated as optimal to treat dermal wounds.

## Figures and Tables

**Table 1:** Clinical studies of cultured adipose tissue-derived stromal cells (ASCs) as treatment of wound healing.

Reference	Study Type	Study Population	Intervention	Follow Up	Results	Complications
Bura et al. 2014	Prospective, non-controlled, non-blinded, non-randomized	Patients with non-healing ischemic ulcers. Age of ulcers was at least 2 weeks (n = 7).	Intervention: 10^8^ of cultured ASCs (0.5 mL) injected intramuscular.	Ulcer healing was determined by measuring the largest diameter of the ulcer, pain was assessed with a VAS score and limb ischemia was assessed by TcPO^2^ with laser Doppler and ABI after 1, 3 and 6 months.	4 patients underwent amputation within 5 months after treatment. Pain was decreased in 3 patients. TcPO^2^ was increased after 6 months as compared to preoperative, except for one patient. *	No complications reported.
Lee et al. 2012	Prospective, non-controlled, non-blinded, non-randomized	Patients with critical limb ischemia and non-healing ulcers or necrosis (n = 12).	Intervention: 5 × 10^6^ of cultured ASCs (0.5 mL) injected intramuscular.	Pain was evaluated with a Wong Baker-FACES rating score, an ABI was measured, walking distances was measured with a treadmill and temperature changes were measured with a thermography after 6 months.	Ulcer healing occurred in 66.7% of the patients. Pain was decreased as compared to the baseline. * Claudication walking distances improved, however maximum walking distance did not (n = 5). * Temperature increased after injection. ** No changes in ABI were noted.	1 mild fever, 1 flu like symptoms, 2 pain, 1 headache.

ASC = adipose derived stromal cells, VAS = visual analogue scale, TcPO^2^ = transcutaneous oxygen pressure, AB = ankle-brachial index. * Results were significant when *p* < 0.05. ** Results were significant when *p* < 0.01.

**Table 2:** Clinical studies of cellular stromal vascular fraction (SVF) as treatment of wound healing.

Reference	Study Type	Study Population	Intervention	Follow Up	Results	Complications
Marino et al. 2013	Prospective, controlled, non-blinded, non-randomized	Patients with peripheral arterial disease and non-healing chronic ulcers of the lower limb (n = 10 vs. n = 10).	Intervention: 3 × 10^5^ of cellular SVF per ml (5 mL) injected at the edge of the ulcers. Control: non-treated	Results were evaluated after 4, 10, 20, 60 and 90 days.	6 of the 10 patients treated with SVF cells showed a complete healing of the ulcer and a decrease of pain. 4 patients treated with SVF cells did not respond. No comparison data between intervention group and control group mentioned.	No complications reported.
Del Papa et al. 2015	Prospective, non-controlled, non-blinded, non-randomized	Patients with digital ulcers. Age of the ulcer was at least 5 months (n = 15).	Intervention: 0.5–1 mL of cellular SVF injected at the base of the fingers.	Time until the wounds were closed was measured. A VAS score for pain, a nail fold video capillary scope for capillary density and echo-Doppler for the RI score were used after 1, 3 and 6 months.	The mean time for ulcers to heal was 4.23 weeks (range 2–7 weeks). No new digital ulcers appeared during the follow-up. VAS score for pain and RI score were decreased after 6 months as compared to preoperative. *** An increase in capillary density was observed after 6 months with respect to the baseline. ***	No complications reported.
Han et al. 2010	Prospective, controlled, single-blinded, non-randomized	Patients with diabetic foot ulcers. Ulcers were non-responsive for at least 6 weeks (n = 26 vs. n = 26).	Intervention: 4 × 10^6^–8 × 10^8^ of cellular SVF in 0.3–0.5 mL of fibrinogen. Co-intervention: debridement, thrombin, Tegaderm™ foil. Control: fibrinogen and thrombin.	Ulcer healing was evaluated by a blinded panel after 8 weeks.	Complete ulcer healing occurred in all patients in the intervention group, while complete ulcer healing occurred in 62% of the patients in the control group. *	No complications reported.
Darinskas et al. 2017	Prospective, non-controlled, non-blinded, non-randomized	Patients with critical limb ischemia and ulcers (n = 6).	Intervention 1: at least 20 × 10^6^ of cellular SVF (20 mL) along the arteries. Intervention 2 (after 2 months): at least 20 × 10^6^ of cellular SVF (20 mL).	Ulcer healing, pain, changes in walking distance as well as ABI were evaluated after 12 months.	5 patients showed clinical improvement, improvement in walking distance, relief of pain and ABI improvement. 1 patient underwent a major amputation. No ulcer recurrence was noted during follow-up.	No complications reported.
Konstantinow et al. 2017	Prospective, non-controlled, non-blinded, non-randomized	Patients with chronic lower limb ulcers. Age of ulcers was at least 6 months (n = 16).	Intervention: cellular SVF (2.54 mL) injected into the border and central area of the ulcer. Co-intervention: Octenisept^®^, debridement, collagen sponge, silicon foil, semipermeable transparent foil.	Reduction in wound size was evaluated until 44 months postoperative (9–44 months). Postoperative pain was evaluated within 2 weeks after treatment.	11 patients showed complete epithelialization within 71–174 days postoperative. Postoperative pain decreased from a mean value of 3.3 (range 1–5, median 3) to a mean value of 0.6 (range 0–3.5, median 0.5).	No complications reported.

SVF = stromal vascular fraction, VAS = visual analogue scale, RI = arterial resistivity index (resistance to blood flow caused by a microvascular bad distal to the measurement site), ABI = ankle-brachial index. * Results were significant when *p* < 0.05. *** Results were significant when *p* < 0.001.

**Table 3:** Clinical studies of lipografting as treatment of wound healing.

Reference	Study Type	Study Population	Intervention	Follow Up	Results	Complications
van Abeelen et al. 2014	Case report	Patient with recurrent leaks from her stoma and skin excoriation.	Intervention: multiple layer lipografting around the stoma. Co-intervention: Tegaderm™ foil.	Results were evaluated after 12 months.	No clinical recurrence occurred.	No complications reported.
Del Berne et al. 2014	Prospective, non-controlled, non-blinded, non-randomized	Patients with Systemic Sclerosis and digital ulcers (n = 9, 15 ulcers). Age of the ulcer was 2–8 months.	Intervention: lipografting at the border of the ulcer. Co-intervention: Iloprost (intravenously), calcium channel blockers, Bosentan, Sildenafil, Aspirin and debridement.	Results were evaluated after 3 months. Another 6 months to 2 years of follow-up was used to evaluated any ulcer recurrence.	10 of the 15 ulcers healed completely in 8 to 12 weeks. In 2 patients (3 ulcers) amputation was needed. In 2 patients, the ulcer size decreased with 50%. All patient, except of 2, reduced their analgesics therapy.	No complications reported.
Caviggioli et al. 2012	Case report	Patient with a posttraumatic leg ulcer.	Intervention: 5 mL of centrifuged adipose tissue. Co-intervention: wound debridement, calcium alginate dressing.	Results were evaluated after 1 week, 2 weeks, 1, 3, 6 and 12 months.	Complete wound closure was obtained after 1 month. Patient satisfaction was excellent.	Not mentioned.
Cervelli et al. 2009	Prospective, controlled, non-blinded, non-randomized	Patients with lower-extremity chronic ulcers and vascular disease (n = 20).	Intervention: lipografting in the bed around the margins of the ulcers. Co-intervention: PRP injection (25 interventions in total). Control: medication-based collagen and hyaluronic acid.	Results were evaluated after 2 and 5 weeks and 3, 6 and 12 months.	16 of the 20 ulcers re-epithelialized after 9.7 weeks on average in the intervention groups compared to 5 of 10 ulcers re-epithelialized in the control group after 8.4 weeks on average. 13 patients needed 1 treatment, 5 patients needed 2 treatments. In 4 patients of the intervention group ulcer recurrence occurred.	Not mentioned.
Cervelli et al. 2010	Prospective, non-controlled, non-blinded, non-randomized	Patients with ulcers or substance loss of the lower limb (n = 30).	Intervention: lipografting in the wounds. Co-intervention: PRP injection, hyaluronic acid.	Results were evaluated every week until 1 month postoperative, then follow-up was done 3, 6 and 12 months postoperative. Biopsies were taken intra-operative and 15 days postoperative.	Complete healing occurred in 57% of the patients after 3 months. Postoperative biopsies showed an increased cell proliferation as compared to intra-operative biopsies. No quantitative data was shown.	2 infections.
Cervelli et al. 2011	Prospective, controlled, non-blinded, non-randomized	Patients with post-traumatic lower extremity ulcers (n = 40).	Intervention 1: SVF enriched lipografting into the bed of the ulcer and peri-lesional. Intervention 2: PRP enriched lipografting into the perilesional area. Control 1: hyaluronic acid into the bed of the ulcer. Control 2: PRP gels into the bed of the ulcer.	Results were evaluated up to 16 weeks postoperative. Biopsies were taken from a small sample size (numbers not mentioned) preoperative and 3, 7 and 16 weeks postoperative.	After 9.7 weeks, re-epithelialization of the wound occurred for 97.9% ± 1.5% for intervention 1, 87.8% ± 4.4% for control 1 *, 97.8% ± 1.5% for intervention 2 and 89.1% ± 3.8% for control 2. * No biopsy comparison data between the four groups was presented.	2 hematoma, 1 infection, 1 edema, 1 edema and infection, 1 edema and hematoma, 1 edema, infection and hematoma.
Klinger et al. 2010	Retrospective, non-controlled	Patients with chronic ulcers within the scar area (n = 8). Non-healed ulcers for 15.4 weeks on average.	Intervention: lipografting in the dermal-subdermal junction of the scar and edge and central region of the ulcer.	Results were evaluated after 2 weeks.	Complete re-epithelialization occurred in all patients after 2 weeks. Patient satisfaction was excellent. Results were stable after 1-year follow-up.	No complications reported.
Stasch et al. 2015	Prospective, non-controlled, non-blinded, non-randomized	Diabetic patients with non-healing lower limb ulcers (n = 25). Age of the ulcer was >2 months.	Intervention: sublesional lipografting into the bottom of the ulcer and the wound edges. Co-intervention: debridement, VAC dressing, sterile silicone wound dressing, Octenisept^®^ and Suprasorb H^®^ plates.	Time until wounds closed and time until wounds closed by 50% was measured. Photographic evaluation of the healing process.	22 of the 25 ulcers healed completely after 68 days on average. Mean wound size reduction of 50% was achieved 4 weeks postoperative. One patient needed a repeated lipografting session and complete wound healing was achieved within another 4 weeks.	No complications reported.

PRP = platelet-rich plasma, SVF = stromal vascular fraction, VAC = vacuum assisted closure. * Results were significant when *p* < 0.05.
